# Generalized multi-channel scheme for secure image encryption

**DOI:** 10.1038/s41598-021-02067-8

**Published:** 2021-11-22

**Authors:** Romil Audhkhasi, Michelle L. Povinelli

**Affiliations:** Ming Hsieh Department of Electrical and Computer Engineering, University of Southern California, Los Angeles, CA 90089 USA

**Keywords:** Nanoscience and technology, Optics and photonics

## Abstract

The ability of metamaterials to manipulate optical waves in both the spatial and spectral domains has provided new opportunities for image encoding. Combined with the recent advances in hyperspectral imaging, this suggests exciting new possibilities for the development of secure communication systems. While traditional image encryption approaches perform a 1-to-1 transformation on a plain image to form a cipher image, we propose a 1-to-n transformation scheme. Plain image data is dispersed across *n* seemingly random cipher images, each transmitted on a separate spectral channel. We show that the size of our key space increases as a double exponential with the number of channels used, ensuring security against both brute-force attacks and more sophisticated attacks based on statistical sampling. Moreover, our multichannel scheme can be cascaded with a traditional 1-to-1 transformation scheme, effectively squaring the size of the key space. Our results suggest exciting new possibilities for secure transmission in multi-wavelength imaging channels.

## Introduction

Advances in the study of optical metamaterials^1,2^ have introduced new possibilities for encoding information in optical waves, in both the spatial^3–6^ and spectral^7–12^ domain. At infrared wavelengths, metamaterials have been designed to encode images in their spatial emission profiles^13,14^. At visible wavelengths, metamaterials have been used to encode images in the form of static^15–22^ or dynamically-modulated^23–27^ holograms. Moreover, metamaterials can be used to create multiplexed holograms on independently measured wavelength channels^28–30^. This capability suggests intriguing new possibilities for spatial and spectral encryption. For example, metasurfaces have been used to visually “hide” an image by distributing its pixels over multiple wavelength^31–33^ or polarization^24^ channels. In this work, the original image was easily recovered by summing over channel outputs. Here, we probe the challenge of *secure encryption*: how can the image information be distributed so that it can only be recovered by an intended recipient?

**Figure 1:**
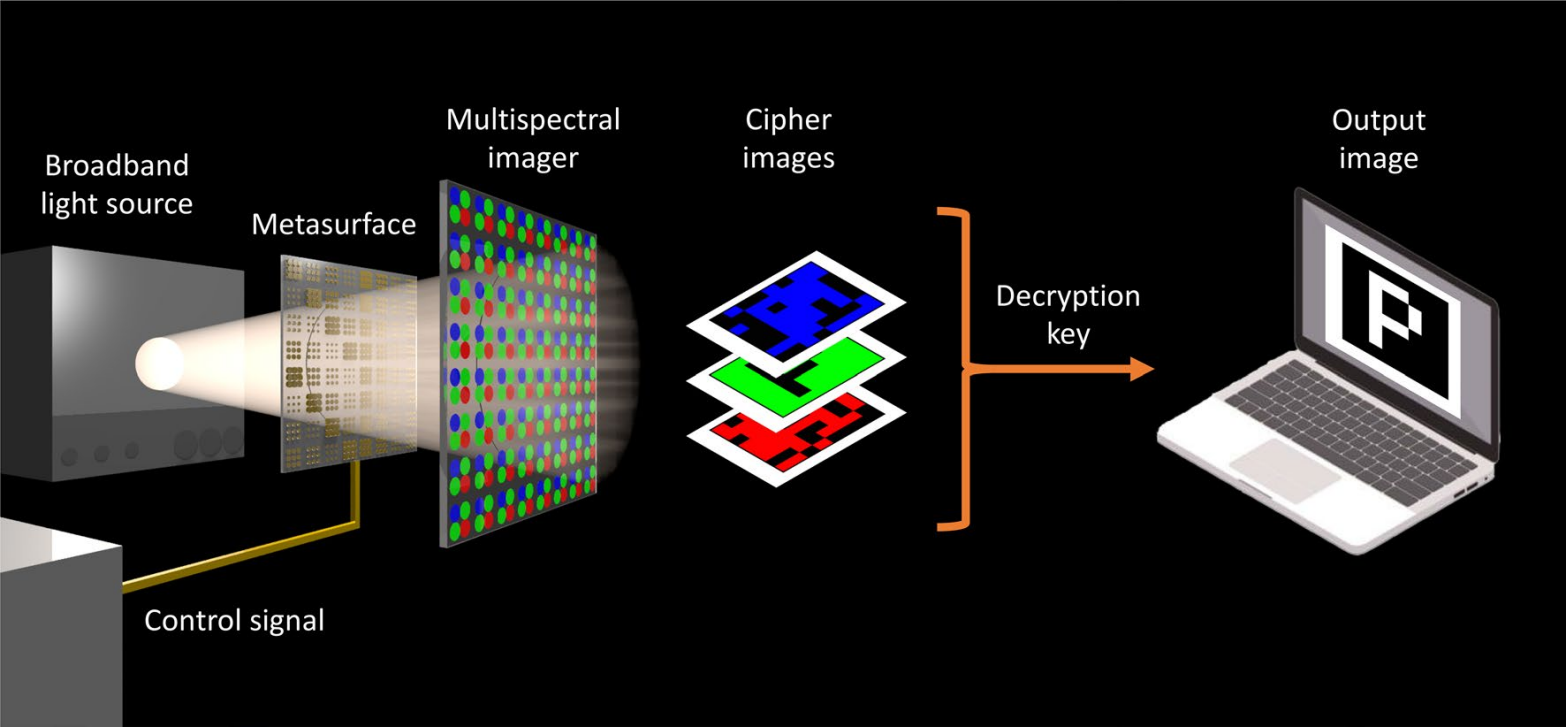
Potential implementation of our encryption scheme. A controllable metasurface (or alternatively, a spatial-light modulator or dynamic hologram) is used to imprint spatial and spectral information onto a broadband light source. To decrypt the image, a recipient must use a multispectral imager to record individual wavelength channels, or *cipher images* and apply a decryption key. A CMOS array will, for example, provide three channels (RGB), while advanced multispectral or hyperspectral imagers can increase the number of channels to 128^43^.

In this work, we define a generalized encryption and decryption algorithm suited to the transmission of image data on multiple wavelength channels. For concreteness, we consider the transmission of images depicting letters chosen from a finite alphabet. The algorithms depend upon possession of a key, which is chosen from a space of all possible keys (*key space*). Unlike traditional image encryption schemes, which transform the image into a single cipher image^34–42^ (1-to-1 transformation), our scheme performs a 1-to-*n* transformation to distribute the image across multiple wavelength channels (see Fig. 1). The images measured on each channel serve as *cipher images*, from which the intended recipient can recover the original image by using a decryption key.

We first show that the size of our key space grows double exponentially in the number of channels, providing security against brute-force attack when the number of channels is greater than 10. We then evaluate the security of the encryption scheme against a more sophisticated attack, one based on statistical sampling of the key space. The security again increases with the number of channels. Crucially, our multi-spectral encryption scheme can be cascaded with traditional 1-to-1 image encryption approaches, effectively introducing a new dimension of security in transmission.

## Results

### Encryption and decryption algorithms

**Figure 2:**
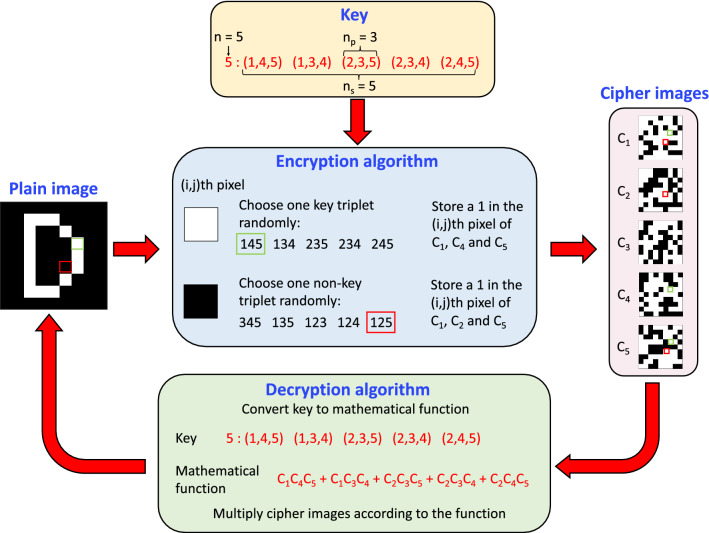
Encryption and decryption algorithms. A 9 × 9 pixel binary image of the letter ‘D’ is input to the encryption algorithm that uses a key to convert it into a set of 5 seemingly random cipher images. The decryption algorithm uses the same key to retrieve the letter ‘D’ image.

We begin by defining general terms relevant to our encryption scheme. The image being encrypted is referred to as a *plain image*. For simplicity, we assume our plain images to be binary, i.e. each pixel has a value of 0 or 1. Figure 2 shows an example plain image, a 9 × 9 letter ‘D’. Here, the black pixels have a value of 0 and the white pixels have a value of 1. The plain image is transformed into a set of *cipher images* using an *encryption algorithm*, a mathematical procedure that depends on the choice of a *key*. Intuitively, the goal of encryption is to “hide” the information present in the plain image. Figure 2 shows the cipher images C_1_ through C_5_ generated using a specific choice of the key. None of the images obviously resembles a ‘D.’ An *output image* is generated by applying a *decryption algorithm* to the cipher images. If the same key is used for decryption as encryption, the output image is the same as the plain image. In general, the number of possible keys (size of the *key space*) should be large enough that an attacker is unlikely to guess the correct key at random.

Our key space is implicitly defined by a set of mathematical decryption functions, where each function is written as a sum of products (SOP) of cipher images. Decryption is performed by operating the SOP function on the cipher images. For images C_1_ through C_n_, the output image is a sum of *n*_*s*_ terms, where each term is a product of *n*_*p*_ non-repeating cipher images. For instance, consider the SOP function shown in the green box in Fig. 2, {C_1_C_4_C_5_ + C_1_C_3_C_4_ + C_2_C_3_C_5_ + C_2_C_3_C_4_ + C_2_C_4_C_5_}. All operations are performed in a pixel-wise fashion. In this case, *n* = 5, *n*_*p*_ = 3, and *n*_*s*_ = 5. The corresponding key is a sequence of integers that represents the SOP function. The integer before the colon indicates the number of cipher images (here, *n* = 5), and the integers following the colon represent the product terms. At a given pixel location, a product term contributes a value of 1 to the output image if and only if all the cipher images in the product term have a 1 at that pixel location. For example, the product term C_1_C_4_C_5_ at the pixel location (4,7) indicated by the green box in Fig. 2 produces the white pixel indicated in the output image.

The first step of the encryption algorithm can be understood as the converse of decryption (blue box in Fig. 2). For ease of reference, we call the five product terms in the example ‘key triplets’. For *n* = 5, one can have at most 10 triplets of non-repeating integers (excluding permutations). The remaining five triplets that do not appear in the key are referred to as ‘non-key triplets’. Each white pixel (value 1) of the plain image is randomly assigned to a key triplet, and a 1 is stored at the same location in its constituent cipher images. For example, the white pixel highlighted by the green box in the letter ‘D’ image is assigned to the triplet (1,4,5), so that cipher images C_1_, C_4_ and C_5_ each have a 1 at their (4,7) pixel locations. This approach uniformly divides the ‘1’ pixels of a plain image among its cipher images, visually disguising the information in the plain image.

The second step of the encryption algorithm introduces “red herring” pixels in each cipher image. This is accomplished by assigning each black pixel (value 0) of the plain image to a randomly-chosen non-key triplet and storing a 1 at the same location in its constituent cipher images. For example, C_1_, C_2_ and C_5_ each have a 1 at their (6,6) pixel locations. The triplet (1,2,5) does not appear in the key, and the plain image has a 0 at this location (highlighted by the red box). The red herring pixels prevent an attacker from deducing the non-zero pixels of the plain image simply by noting the locations of any non-zero pixels in the cipher images. Moreover, since all pixels of the plain image are mapped to either a key triplet or a non-key triplet, a simple sum of all cipher images yields a uniform image, devoid of information. This point is illustrated with an example in the next section.

We note that repeated applications of the encryption algorithm to the same plain image can yield a different set of cipher images, even when the key is held fixed. This is due to the element of randomness in the algorithm that occurs when assigning pixels to key and non-key product terms.

### Enabling security against a brute-force attack

**Figure 3:**
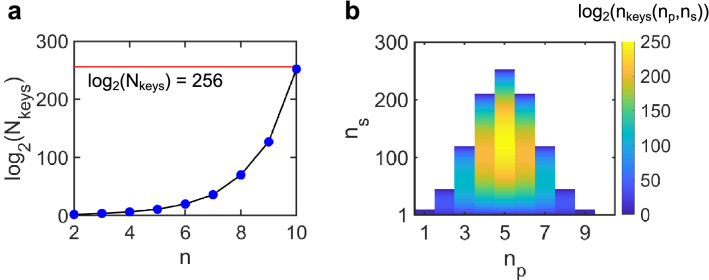
Size of the key space. **(a)** Variation of log_2_(*N*_*keys*_) with *n*. The solid red line shows the AES limit of *N*_*keys*_ = 2^256^ for symmetric encryption. (**b)** Variation of log_2_(*n*_*keys*_) with *n*_*p*_ and *n*_*s*_ for *n* = 10.

For the encryption algorithm to be resistant to a brute-force attack, the key space should be large enough that an attacker cannot manually test all the possible keys. Given the number of cipher images, we can determine the total number of possible keys, *N*_*keys*_ by combinatorics. This calculation is presented in the Methods section. Figure 3a shows a plot of log_2_(*N*_*keys*_) versus *n*. For reference, the maximum key space size of 2^256^ for practical AES symmetric encryption is shown by the solid red line. It can be observed that the total number of keys increases double-exponentially with *n* and becomes nearly equal to the AES limit for *n* = 10. This indicates that encrypting a plain image into more than 10 cipher images would render a brute force attack infeasible.

**Figure 4:**
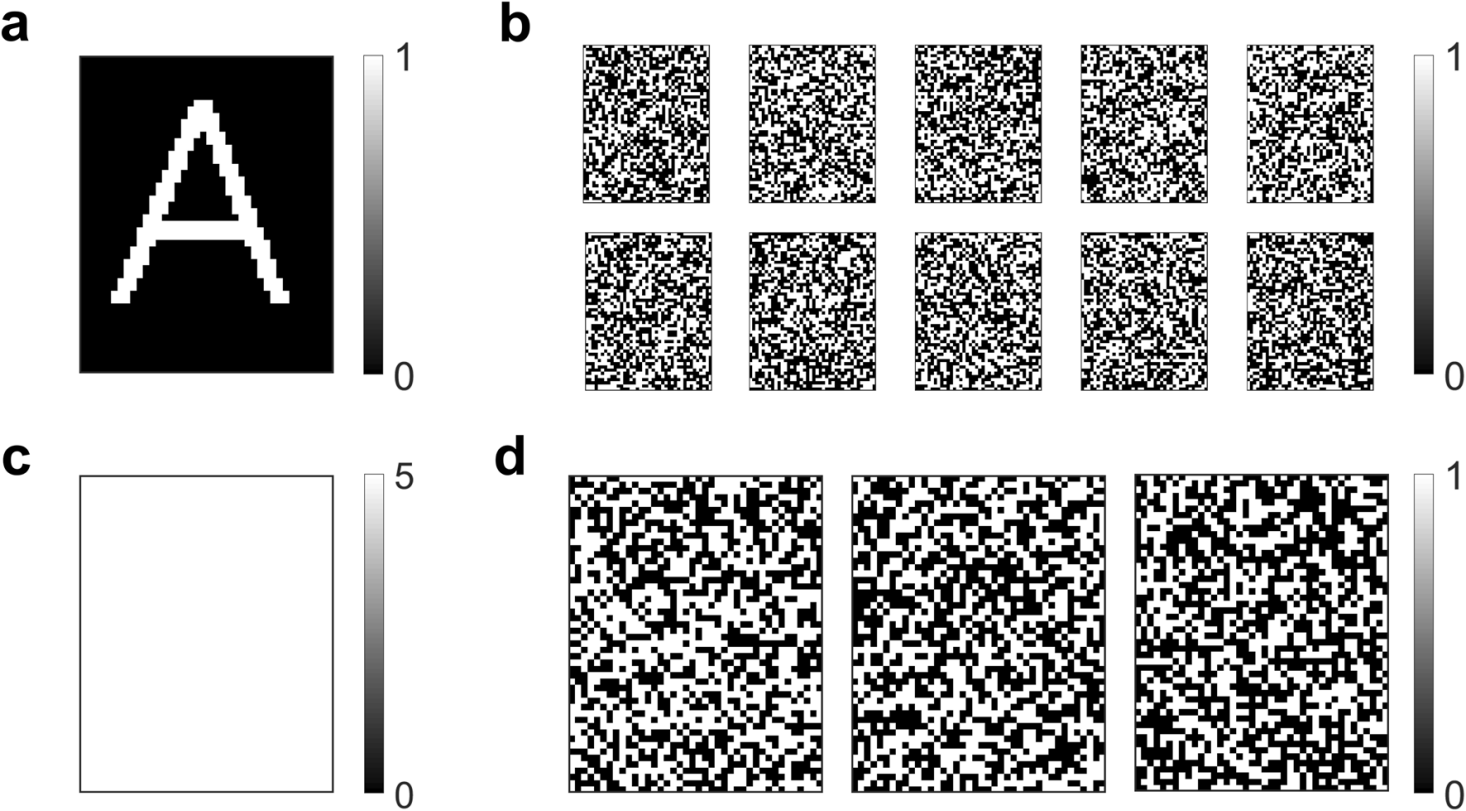
Encryption using optimal system parameters. **(a)** A 50 × 40 pixel binary image of the letter ‘A’. (**b)** Cipher images generated by encrypting the letter ‘A’ image using a key with *n* = 10, *n*_*p*_ = 5 and *n*_*s*_ = 126. (**c)** Image representing the sum of all cipher images. (**d)** Images generated by attempting decryption using three incorrect keys with *n* = 10, *n*_*p*_ = 5 and *n*_*s*_ = 126.

We denote the number of keys for fixed *n*, *n*_*p*_ and *n*_*s*_ as *n*_*keys*_. Figure 3b shows the variation of log_2_(n_keys_*)* with *n*_*p*_ and *n*_*s*_ for *n* = 10. Here *n*_*s*_ ranges from 1 to *C*(10,*n*_*p*_) and *n*_*p*_ ranges from 1 to 10. It can be observed that *n*_*keys*_ is maximum for ${n}_{p}= \left|\!{\underline{\,\,\,\,}}\right.  10/2 {\left. {\underline{\,\,\,\,}}\!\right|}=5$ and ${n}_{s}= \left|\!{\underline{\,\,\,\,}}\right.  C(\mathrm{10,5})/2 {\left. {\underline{\,\,\,\,}}\!\right|}=126$, where $\left|\!{\underline{\,\,\,\,}}\right.  x {\left. {\underline{\,\,\,\,}}\!\right|}$ denotes the greatest integer less than or equal to *x*. In general, one should choose ${n}_{p}= \left|\!{\underline{\,\,\,\,}}\right.  n/2 {\left. {\underline{\,\,\,\,}}\!\right|}$ and ${n}_{s}= \left|\!{\underline{\,\,\,\,}}\right.  C(n,{n}_{p})/2 {\left. {\underline{\,\,\,\,}}\!\right|}$, which maximizes the size of the key space (see Fig. 4 for a numerical example with n = 10).

To illustrate the security of the optimized system against brute-force attacks, consider a 50 × 40 pixel binary image of the letter ‘A’ (Fig. 4a) that has been encrypted using a key with *n* = 10, *n*_*p*_ = 5 and *n*_*s*_ = 126. The resulting cipher images are displayed in Fig. 4b. As can be seen, simple visual inspection of the cipher images does not reveal any meaningful information about the plain image. Moreover, a sum of all cipher images results in a uniform intensity image with all pixel values equal to 5 (Fig. 4c).

We assume that the attacker has access to the cipher images and the system parameters that were used for encryption (*n*, *n*_*p*_, and *n*_*s*_). We further assume that the attacker has knowledge of the encryption and decryption algorithms but does not have access to the encryption key. So, he resorts to randomly trying out a small number of keys with *n* = 10, *n*_*p*_ = 5 and *n*_*s*_ = 126 and visually inspecting the output images to guess the encrypted plain image. Figure 4d shows the output images for three such keys, none of them being the original key used for encryption. Here again, it is difficult to gather any information about the plain image by simply looking at the output images. Therefore, one would expect that an attacker relying solely on visual inspection would have a very low probability of recovering a plain image encrypted using our scheme.

### Enabling security against more sophisticated attacks

Next, we evaluate the security of our encryption scheme in a scenario where an attack more sophisticated than simple visual inspection is used to recover a plain image. In the example of Fig. 4, none of the incorrect keys tried yielded an output image that obviously resembled the plain image (Fig. 4d). However, one might ask whether there is more subtle information contained in the output images obtained from incorrect keys. A more sophisticated attacker might therefore go beyond visual inspection to calculate a similarity score with a known set of possible plain images.

We consider the problem of transmitting messages written using a four-letter alphabet. The alphabet comprises of 50 × 40 pixel binary images of the letters ‘A’, ’B’, ’C’ and ‘D’. Let’s assume that the letter ‘A’ needs to be transmitted and has been encrypted using a key with *n* = 10, *n*_*p*_ = 5 and *n*_*s*_ = 126. An attacker intercepts the transmission channel and gains access to the cipher images. We assume that the attacker is familiar with the encryption and decryption algorithms but does not have access to the key that was used. Since there are ten cipher images, he guesses that *n* = 10.

**Figure 5:**
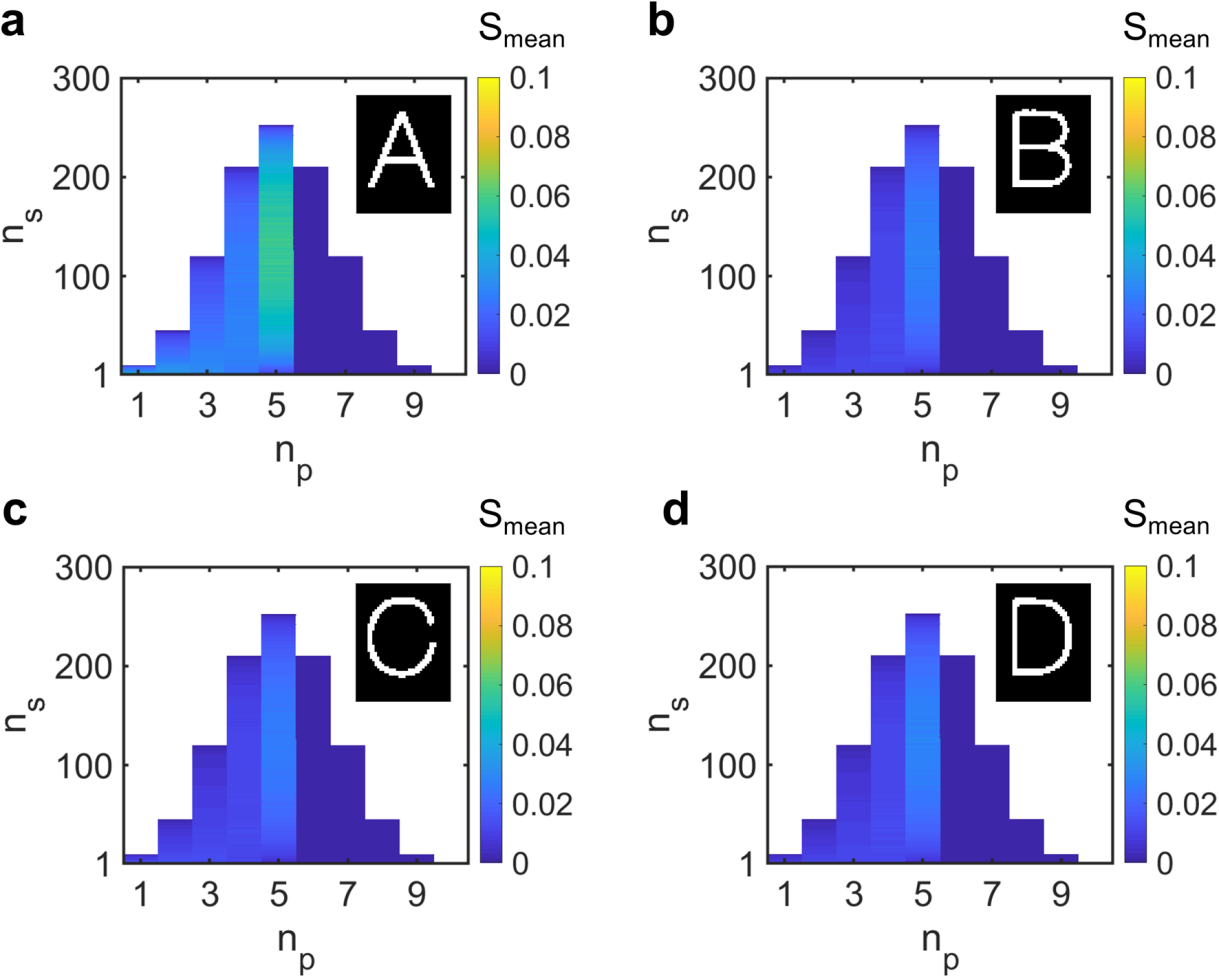
Sophisticated attack based on statistical sampling of key space. (**a–d)** Variation of *S*_*mean*_ for encrypted letter ‘A’ with *n*_*p*_ and *n*_*s*_ calculated with respect to letters ‘A’ through ‘D’. *S*_*mean*_ for each *n*_*p*_ and *n*_*s*_ is equal to the mean similarity score of output images obtained by operating 1000 randomly selected keys on the cipher images.

As discussed previously, the size of the key space for *n* = 10 is large enough to make it infeasible for the attacker to try out all possible keys. To get around this problem, the attacker constructs a randomly-selected sample set of 1000 keys for each *n*_*p*_ and *n*_*s*_. He uses these keys to generate 1000 output images and computes their mean similarity score, *S*_*mean*_ with respect to the four letters. The definition of similarity score is presented in the Methods section. The score *S*_*mean*_ with respect to the letters ‘A’ through ‘D’ is displayed as a function of *n*_*p*_ and *n*_*s*_ in Fig. 5a through d. We note that in a practical situation, the attacker would stop traversing the key space as soon as he hits the right key. We assume that this does not happen in this situation as the probability of finding the right key is very low (~ 1/10^74^).

From Fig. 5, one can observe that for *n*_*p*_ ≤ 5, the mean scores with respect to letter ‘A’ are in general higher than those for all the other letters. In particular, *S*_*mean*_ for letter ‘A’ takes its maximum value close to *n*_*p*_ = 5 and *n*_*s*_ = 126, which are the parameters used for encryption. Therefore, the attacker will be able to guess the encryption parameters and the encrypted letter by simply looking at the mean score colormaps for the four letters.

In addition, it can be observed that the mean scores for all keys with *n*_*p*_ > 5 are equal to zero. Since the letter ‘A’ image was encrypted using a key with *n*_*p*_ = 5 and *n*_*s*_ = 126, each of its ‘1’ pixels is stored in one of the 126 groups of five channels. This implies that only five of the ten cipher images can store a ‘1’ at any given pixel location. Multiplying more than five cipher images would result in a complete cancelation of pixel values and generate an image with all pixels equal to 0. This happens when evaluating the decryption function for keys with *n*_*p*_ > 5. Decryption using such keys results in all-zero images that have a similarity score of 0 with respect to all the four letters.

**Figure 6:**
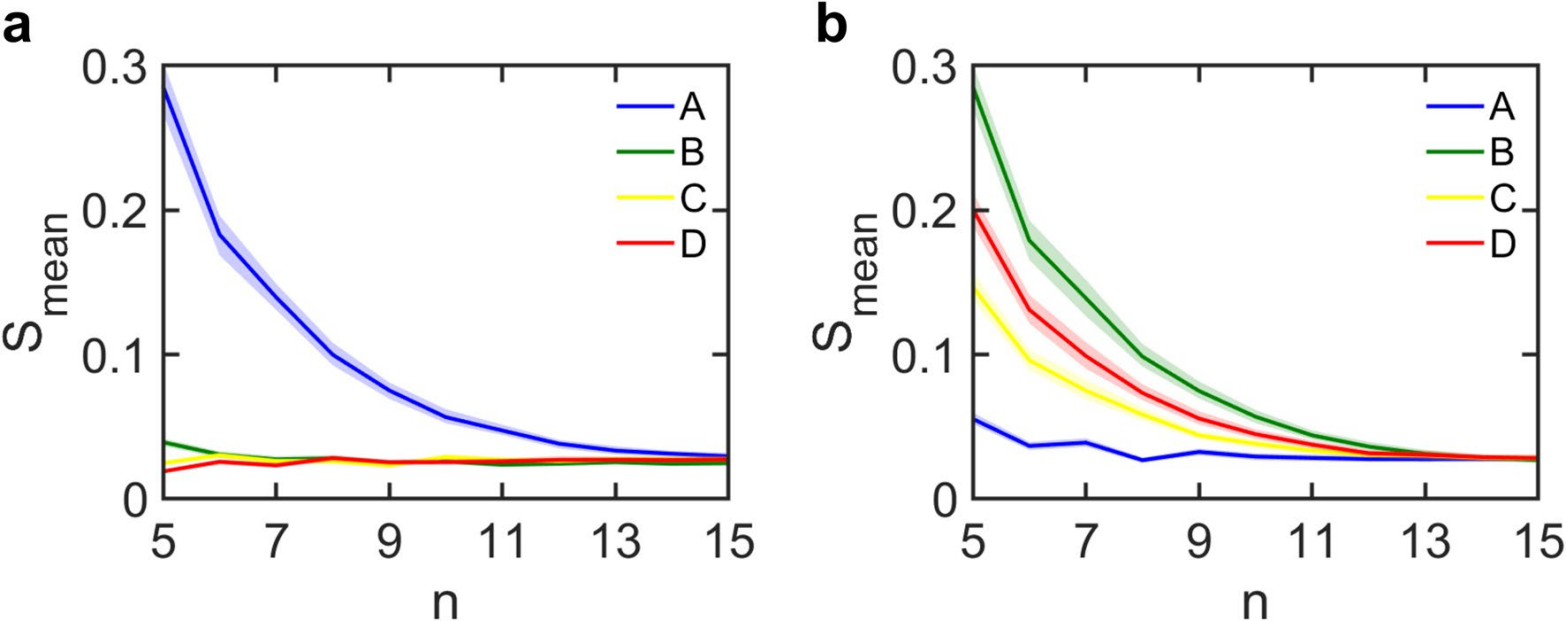
Improving system security against sophisticated attack. Variation of mean score *S*_*mean*_ with *n* calculated with respect to letters ‘A’ through ‘D’ for encrypted letter (**a)** ‘A’ and (**b)** ‘B’. Solid lines indicate average S_mean_ over 1000 trails with 1000 keys each, and colored bands represent the corresponding error bounds.

In order to make it difficult for the attacker to identify the encrypted letter, we must decrease the difference in *S*_*mean*_ calculated with respect to the four letters. One way to accomplish this is to increase *n*. Figure 6a presents the variation of *S*_*mean*_ with *n* for letter ‘A’ encrypted using ${n}_{p}= \left|\!{\underline{\,\,\,\,}}\right.  n/2 {\left. {\underline{\,\,\,\,}}\!\right|}$ and ${n}_{s}= \left|\!{\underline{\,\,\,\,}}\right.  C(n,{n}_{p})/2 {\left. {\underline{\,\,\,\,}}\!\right|}$. Since S_mean_ tends to be higher close to the encryption parameters, we only present its value at ${n}_{p}=  \left|\!{\underline{\,\,\,\,}}\right.  n/2 {\left. {\underline{\,\,\,\,}}\!\right|}$ and ${n}_{s}= \left|\!{\underline{\,\,\,\,}}\right.  C(n,{n}_{p})/2 {\left. {\underline{\,\,\,\,}}\!\right|}$ for each *n*. The solid lines represent the average S_mean_ computed over 1000 trials with 1000 samples each, while the colored bands represent the corresponding error bounds.

One can notice that for small values of *n*, the scores with respect to ‘A’ are significantly larger than those with respect to the other letters. As *n* increases, S_mean_ with respect to ‘A’ reduces while it remains nearly constant for the other letters. For *n* $\ge$ 14, the error bounds on S_mean_ for ‘A’ start to overlap with those for ‘C’ and ‘D’. This implies that encrypting ‘A’ into 14 or more cipher images will make it difficult for the attacker to identify it on the basis of similarity scores. One may note that the value of *n* needed to ensure security in this case is higher than that required to prevent a brute-force attack (*n* = 10). In general, the number of cipher images (*n*) required to defend against a sophisticated attacker who uses a randomly-selected key set is larger than for a brute-force attacker. However, provided *n* is chosen large enough, the system will remain secure.

Even though the analysis presented thus far is for encrypted letter ‘A’, the conclusion remains the same for all letters in the alphabet. To validate this, we present the variation of *S*_*mean*_ with *n* for encrypted letter ‘B’ in Fig. 6b. Here again, the scores with respect to letter ‘B’ are higher for small values of *n* and reduce as *n* increases. One can also note that the scores with respect to letters ‘C’ and ‘D’ in Fig. 6b are close to those with respect to letter ‘B’ due to the similarity in the shapes of these three letters. For *n* $\ge$ 13, the error bounds on S_mean_ for ‘B’ start to overlap with those for the other letters. Therefore, from Fig. 6a,b, one can conclude that choosing an *n* $\ge$ 14 makes it difficult for the attacker to identify messages written using a combination of ‘A’ and ‘B’. A similar calculation can be done for the letters ‘C’ and ‘D’ to determine a lower bound on *n* for the entire system. Similar conclusions are obtained for the case in which an attacker decides to use maximum scores instead of mean scores. In this situation, the error bands on scores are much broader and the lower bounds on *n* for secure encryption of letters ‘A’ and ‘B’ are 12 and 11, respectively.

### Experimental demonstration

**Figure 7:**
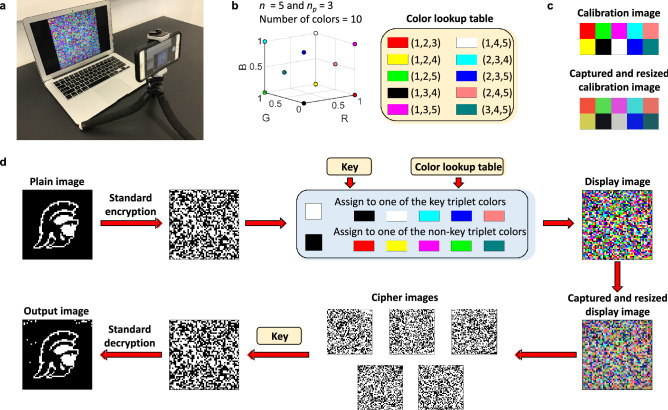
Table-top demonstration of our encryption scheme. **(a)** A computer screen displays an image which is captured by a phone camera. (**b)** Triplets of channels corresponding to *n* = 5 and *n*_*p*_ = 3 are mapped to 10 colors in the RGB space to generate a color lookup table. (**c)** A calibration image comprising of all 10 colors displayed on the computer screen is captured and resized to the original resolution, to account for color distortion. (**d)** A 50 × 50 pixel binary image of the USC Trojan logo is converted to a random display image using a key and the color lookup table. The display image is captured by a camera, resized and converted into a set of 5 cipher images using the calibrated color lookup table. A key is then used to convert the cipher images into an output image.

To illustrate the utility of our encryption scheme, we conduct a table-top demonstration using a color display and a color camera (Fig. 7a). While a true *n*-channel encryption scheme (as described above) requires independent control over transmission and detection at *n* distinct wavelengths, we can emulate the full behavior using a simple RGB system (Fig. 7b) with calibration (Fig. 7c). We demonstrate our encryption algorithm within a cascaded encryption scheme (see ‘Methods’ for details). A plain image is first encrypted using a standard scheme to produce an apparently random image. Using the key, the white and black pixels are randomly assigned to the key triplet and non-key triplet colors, respectively (Fig. 7d) to produce the display image. We capture the display image on the camera and use the color lookup table to recover the output image, as shown. The output image shows high fidelity with respect to the original plain image with a similarity score of 0.98. This simple demonstration shows the robustness of our encryption system to noise.

## Discussion

The demonstration of Fig. 7 illustrates how our multi-spectral scheme can be used to add an “extra dimension” of security to image encryption. In the example above, we cascaded standard and multi-spectral encryption schemes. To successfully decrypt the resultant image, the receiver must correctly guess both the standard key K_1_ and the multi-spectral key K_2_. Suppose the standard scheme uses a 256-bit key chosen from a key space size of 2^256^. For a multi-spectral scheme with *n* > 10, the cascaded key space is larger than (2^256^)^2^. The multi-spectral scheme we describe thus effectively squares the key space size. We conclude that our approach can provide an extra dimension for secure encryption, one which can leverage emerging technologies for multi-wavelength transmission and imaging.

In summary, we proposed an encryption scheme based on pixel multiplexing for transmission of images across multiple wavelength channels. Our encryption algorithm divides the pixels of a given plain image into multiple, seemingly random cipher images. Decryption by the intended recipient is performed by using a key to convert the cipher images into meaningful information. We considered a generalized key space based on mathematical decryption functions, each written as a sum of products of cipher images. Using combinatorics, we showed that encryption of a given plain image into more than 10 channels ensures security against a brute-force attack. We also considered a more sophisticated attack; one in which an attacker uses mean similarity scores of randomly chosen samples of the key space to extract information about the plain image. For a 50 × 40 pixel image, we showed that encryption remains secure as long as more than 14 channels are used.

While this work uses RGB display and imaging to emulate a 5-channel scheme, we find that increasing the number of channels leads to increased noise levels and decryption errors. True implementation of *n*-channel encryption and decryption will require independent control of transmission and detection on n separate wavelength channels. To this end, the continued development of tunable metasurfaces, paired with multi- and hyper-spectral imagers, will illustrate the true potential of the proposed encryption method.

## Methods

### Calculation of total number of keys for fixed *n*

For a given *n*, the number of cipher images in each product term (*n*_*p*_) can range from 1 to *n*. The total number of distinct product terms of *n*_*p*_ cipher images is given by $C\left(n,{n}_{P}\right)\equiv \frac{n!}{{n}_{p}!\left(n- {n}_{p}\right)!}$. Therefore, for fixed *n* and *n*_*p*_, the number of product terms in the decryption function (*n*_*s*_) can vary from 1 to *C(n,n*_*p*_). The number of keys for fixed *n*, *n*_*p*_ and *n*_*s*_ is then equal to ${n}_{keys}\left(n,{n}_{p},{n}_{s}\right)=C(C\left(n,{n}_{p}\right),{n}_{s})$*.* Summing over all *n*_*p*_ and *n*_*s*_, the total number of keys for fixed *n* is given by:

1 $${N}_{keys}\left(n\right)= \sum_{{n}_{s}=1}^{C(n,{n}_{p})}\sum_{{n}_{p}=1}^{n}{n}_{keys}(n,{n}_{p},{n}_{s})$$

### Definition of similarity score

The similarity score *S* for an image *M’* with respect to an image *M* is given by:

2 $$S= \left|\frac{cov(M,M{^{\prime}})}{cov(M,M)}\right|$$

Here *cov(M’,M)* refers to the covariance of *M’* and *M*. In our analysis, *M* is a binary image depicting a letter while *M’* is an output image obtained by operating a key on the cipher images possessed by the attacker. We normalize *M’* by its maximum value to ensure that none of its pixels take a value greater than 1. As a result, the similarity score takes a value between 0 (totally dissimilar images) and 1 (*M’* = *M*).

### Experimental demonstration

The original plain image is first converted to a random image by performing an XOR operation with a 50 × 50 pixel one-time pad. The resulting image is converted into a display image sing the color lookup table created in Fig. 7b. The white pixels of the image are assigned randomly to one of the key triplet colors while the black pixels are assigned to the non-key triplet colors. The resulting display image is captured by a color camera and resized to the original resolution of 50 × 50 pixels. We use the calibrated color lookup table (Fig. 7c) to determine the product term corresponding to each pixel of the display image and retrieve the cipher images. These cipher images are operated upon by the decryption key to retrieve the intermediate image which is converted to an output image by performing an XOR with the one-time pad.

## Data Availability

The data used in this study is available from the authors upon reasonable request.
